# Transposable Elements as Stress Adaptive Capacitors Induce Genomic Instability in Fungal Pathogen *Magnaporthe oryzae*

**DOI:** 10.1371/journal.pone.0094415

**Published:** 2014-04-07

**Authors:** Sonia Chadha, Mradul Sharma

**Affiliations:** 1 Nuclear Agriculture and Biotechnology Division, Bhabha Atomic Research Centre, Mumbai, India; 2 Astrophysical Sciences Division, Bhabha Atomic Research Centre, Mumbai, India; Woosuk University, Republic of Korea

## Abstract

A fundamental problem in fungal pathogenesis is to elucidate the evolutionary forces responsible for genomic rearrangements leading to races with fitter genotypes. Understanding the adaptive evolutionary mechanisms requires identification of genomic components and environmental factors reshaping the genome of fungal pathogens to adapt. Herein, *Magnaporthe oryzae*, a model fungal plant pathogen is used to demonstrate the impact of environmental cues on transposable elements (TE) based genome dynamics. For heat shock and copper stress exposed samples, eight TEs belonging to class I and II family were employed to obtain DNA profiles. Stress induced mutant bands showed a positive correlation with dose/duration of stress and provided evidences of TEs role in stress adaptiveness. Further, we demonstrate that genome dynamics differ for the type/family of TEs upon stress exposition and previous reports of stress induced MAGGY transposition has underestimated the role of TEs in *M. oryzae*. Here, we identified Pyret, MAGGY, Pot3, MINE, Mg-SINE, Grasshopper and MGLR3 as contributors of high genomic instability in *M. oryzae* in respective order. Sequencing of mutated bands led to the identification of LTR-retrotransposon sequences within regulatory regions of psuedogenes. DNA transposon Pot3 was identified in the coding regions of chromatin remodelling protein containing tyrosinase copper-binding and PWWP domains. LTR-retrotransposons Pyret and MAGGY are identified as key components responsible for the high genomic instability and perhaps these TEs are utilized by *M. oryzae* for its acclimatization to adverse environmental conditions. Our results demonstrate how common field stresses change genome dynamics of pathogen and provide perspective to explore the role of TEs in genome adaptability, signalling network and its impact on the virulence of fungal pathogens.

## Introduction

Among fungi, filamentous fungal pathogens are the most devastating agents to crops and cause serious animal and human diseases [1]. Their enormous genetic diversity poses a challenge to develop durable strategies for pathogen management [2]. Exposures to environmental cues exert evolutionary pressure, leading to faster mutation rates, adaptation and survival of these organisms [3]–[4]. Impacts of adaptive changes are evident by tremendous increase in pathogen's population [1]. Adaptive genes such as repetitive DNA are subjected to stronger selective pressure than other genomic regions [5]. Repetitive DNA can be classified as tandemly arrayed (microsatellites, minisatellites and telomeres) and interspersed repeats (transposable elements and processed psuedogenes). Transposable elements (TEs) are abundant in eukaryotes and their prevalence indicates important role of TEs in genome biology [6]. TEs are able to move about the host genome and insert into a host's DNA through either cut-and-paste (DNA or Class II transposons) or copy-and-paste mechanisms via RNA intermediates (Retro or Class I transposons). One might expect that TEs can participate in adaptation by influencing fitness and evolutionary potential of their hosts through insertional mutagenesis, disrupted or enhanced gene expression or gross chromosomal rearrangements [7]. The prolonged habitation of TEs in fungi leads to various kinds of interactions with the remaining genome. Exploring the contribution of TEs in the genome dynamics under environmental cues is imperative to understand fungal adaptation and survival. Genetic variations at these candidate loci might have profound influence on populations, allowing them to persist under changing conditions [8]. Despite its central significance for fungal pathogenicity, one question still poorly understood is how pathogenic fungi alter their genomes for its adaptation under selective pressures.

Stress-inducible mutations can potentially accelerate adaptive evolution in populations [9]. The phytopathogenic fungus *Magnaporthe oryzae* is a particularly good model to analyze the contribution of stress on adaptive evolution. Its high genomic instability and associated factors have been a topic of debate [10]–[12]. The pathogen causes one of the most devastating diseases of rice worldwide [13]. Due to its economic importance, *M. oryzae* was the first among the plant pathogenic fungi with a decoded genome [14]. The genome of *M. oryzae* is rich in repetitive DNA, where, a 9.7% of the genome is made up of repetitive DNA, a significant portion of which is derived from transposable elements. *M. oryzae* has one of the highest-quality annotations of TEs among pathogenic fungi. DNA transposons make up approximately 2% of the *M. oryzae* genomic sequences, whereas 1.6% of the genomic sequences are comprised of non-LTR retroelements (LINEs and SINEs). The class of LTR-retrotransposons forms 3.8% of the genomic DNA [14]–[15]. Due to TEs abundance, these genomic components have been employed for genetic variability studies among *M. oryzae* isolates [16]–[17]. However, the role played by these elements in the evolution of M. oryzae has not been thoroughly explored.

High genomic instability and genetic diversity of *M. oryzae* leads to early breakdown of blast resistance in rice varieties, posing a constant challenge for the rice breeders. In *M. oryzae* field isolates, Pot3 transposon was detected within coding and promoter regions of *AVR-Pita* gene, suggesting TEs role in instability of avirulence genes (*AVR*) [18]–[19]. Transposition of *M. oryzae* MGL element was found in *ACR1*, a gene controlling conidiophores development [20], whereas TE MINE was located in avirulence gene *ACE1* [21]. Further, TEs were also speculated to cause genomic variations among *M. oryzae* isolates [17], [22]. In spite of the importance of blast disease and available information on *M. oryzae* TEs, knowledge about how and which genetic factors will affect genomic stability and diversity under changing environmental conditions is inadequate and needs further investigation.

In ecologies, the genomic host is under constant selective pressures. The mutational potential due to repetitive DNA can be very valuable in generating occasional fitter mutants and potentially accelerating adaptive evolution. Earlier studies explained TEs based comprehensive view of the complex evolutionary forces in shaping the eukaryotic genome by using high throughput sequencing [23]. However, these strategies are rather cumbersome, costly and difficult to optimize. As a result, numbers of TEs analyzed per genome were restricted; limiting their large scale applications and understanding in the scientific community. The activity of TEs is mostly assessed by their transcriptional activity or indirectly observing the phenotypic changes caused by transposition [24]. Therefore, an experimental difficulty is associated with transposon research where analysis of the direct and accurate changes at the genomic level is required. To overcome these problems, here we used PCR based approach where diverse and large family of *M. oryzae* transposable elements were targeted to investigate stress induced genome dynamics and instability. In past, molecular assays have been informative to detect induced genetic alterations in *Daphnia magna* [25], cyanobacteria [26] and plants [27], although these assays were based on random genomic targets. However, for fungal pathogens, studies with focus on the environmental effects on genome dynamics and population structure of pathogen are rather limited.

For our study, we selected temperature stress as it is one of the most common stressor under field conditions or upon migration of pathogen to new environments. In addition, copper stress was selected as the usage of copper based fungicides is a common practice in fields to tackle fungal plant pathogens. Here, we elucidate that these environmental cues induce local bursts of TEs, generating new strains of the fungal pathogen. Further, we identify LTR-retrotransposon Pyret as a major player in reshaping the genome and illustrate its applicability for population studies. We also present evidences that stress induced genome dynamics does not necessarily respond to a feature common to all stresses but they can discriminate among stress signals. Furthermore, we identify stress sensitive spots of *Magnaporthe* genome. These results allow us to postulate that transposable elements as stress adaptive capacitors induce genomic instability in *M. oryzae*. This study is significant to unveil the understanding of how, when and where genome changes occur, thus providing insight into dynamical forces shaping the pathogen genome and its impact on fitness and survival.

## Results

### Stress adaptive capacitance of *M. oryzae*

**Figure 1:**
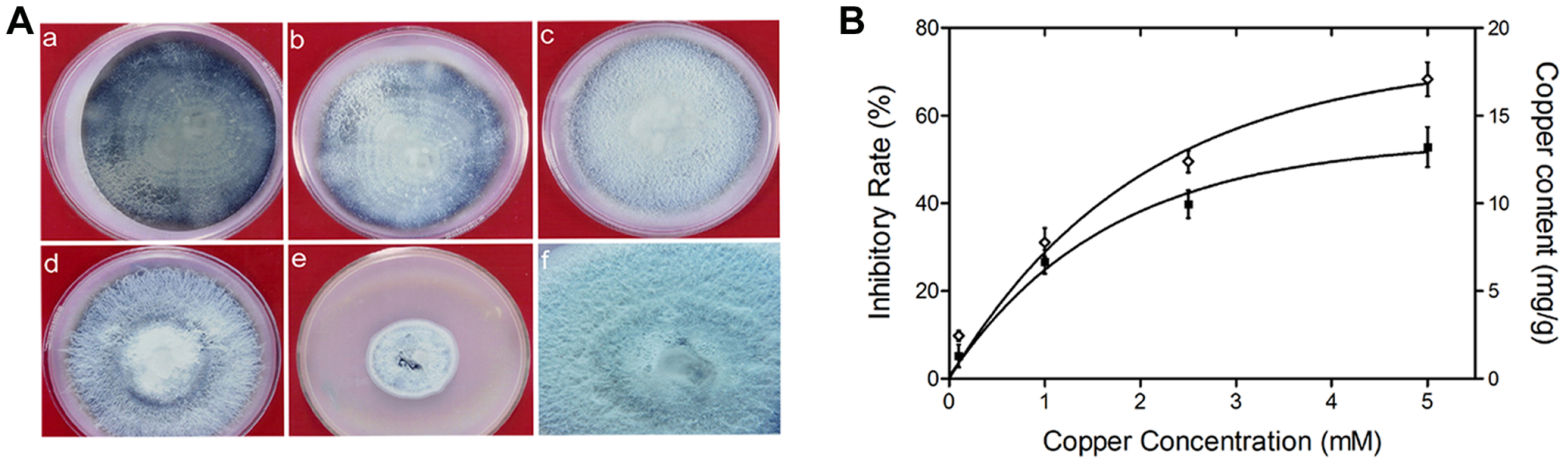
*M. oryzae* show tolerance towards copper stress. A) Prolonged effects of copper stress on fungal growth and phenotype was studied by growing *M. oryzae* cells in presence or absence of copper for 7 days. A regime of copper concentrations was used as shown in a-e which represents 0, 0.1, 1.0, 2.5 and 5.0 mM copper concentrations. Fungal cells showed dense fungal growth and change in hyphal color from gray to white with increasing copper concentration. Dense aerial hyphae were observed in presence of 2.5 mM copper (f). B) Effect of copper on *M. oryzae* growth and its uptake was determined. To estimate, copper acquisition and its effect on fungal growth, 4 days old *M. oryzae* cultures were exposed to copper (0–5 mM) for 18 h. Percent growth inhibitory rate was calculated by comparing growth of stress exposed samples with control (untreated) sample. Inhibitory rate and copper acquisition by *M. oryzae* showed positive correlation (r = 0.97).

To determine the prolonged and continuous effects of copper stress on fungal growth and phenotype, *M. oryzae* cultures were grown on PDA plates in absence or presence of copper (0.1, 1.0, 2.5 and 5.0 mM) for 7 days (Figure 1A). These concentrations are environmentally relevant as found in rice growing soils. The phenotypic and radial growth assays showed dense fungal growth of *M. oryzae* cultures on plates containing 0.1 to 2.5 mM Cu concentrations. Exposure of fungal cultures to 0.1 and 1.0 mM of copper concentrations didn't affect the growth and hyphae color (gray) of *M. oryzae*. Increase in copper concentration to 2.5 mM resulted in the reduced radial growth with dense aerial hyphae. In Cu 5.0 mM samples, fungal growth was reduced by 36% as compared to untreated sample. These findings suggest that *M. oryzae* cells can resist prolonged exposure to environmentally relevant copper concentrations (Figure 1A).

Before evaluating the effect of copper on genome dynamics, we confirmed the uptake of copper by *M. oryzae* cells and its impact on growth. Accumulation of Cu content by *M. oryzae* cells was investigated as a function of initial Cu concentration. The estimated total Cu uptake (mg Cu per g biomass) showed an increase with increasing Cu concentrations (Figure 1B). Even at the lowest copper concentration (0.1 mM), accumulation was observed. A considerable massive uptake was observed at the highest copper concentration (5 mM). A significant correlation (r = 0.98; p<0.05) was observed between the copper acquisition by *M. oryzae* cells and different copper concentrations. The impact of Cu stress on *M. oryzae* growth was determined and expressed as percent growth inhibitory rate (Figure 1B). The copper treated samples (0.1, 1.0, 2.5 and 5.0 mM) inhibited fungal growth by 5%, 26%, 40% and 53% respectively. The IC_50_, the concentration of copper that reduces 50% growth rate at a given concentration was calculated as 2.65 mM. Our results based on copper acquisition, tolerance and its effect on fungal growth suggested adaptive capacity of *M. oryzae* towards copper. Since fungal growth for Cu samples (0.1–2.5 mM) was found below IC_50_ values, these concentrations were selected to evaluate copper effects on genome dynamics and stability.

#### Stress induced genome dynamics

To evaluate stress induced genetic variations, *M. oryzae* culture was exposed to Cu (0.1–2.5 mM) and thermal stress (42°C for 1–3 h). DNA profiles of control and stress exposed fungal samples were generated using outward primers designed to anneal DNA sequences of *M. oryzae* transposable elements MAGGY, Pyret, Grasshopper, Mg-SINE, MINE, MGLR3, Pot3 and Pot2 (Table 1). TEs can potentially integrate in either orientation, enabling the finding of members of a TE family as head-to-head, head-to-tail and tail-to-tail. To ensure the reproducibility, two biological replicates were used and all PCR amplifications were repeated at least twice. Evidences of genomic changes were recorded as variations in loss of normal bands, appearance of new bands and change in band intensity following the stress exposure as compared to untreated control culture. Genotyping profiles of samples subjected to stress produced variable and distinct patterns as compared to control. DNA profiles for the positive control (untreated) sample was found significantly different (p<0.05) from both copper (1.0 and 2.5 mM) and heat shock treated samples (2 and 3 h) using the Dunnett's multiple comparison test (Table S1). DNA profiles of Pyret and Pot2 obtained for control and stress exposed samples are shown in figure S1. Genotyping data showed Pyret generated highest number of mutant bands upon stress exposure and genetic alterations were dependent on the type of DNA targets and primer sequences (Table S2). Dendrogram was constructed using genotyping data obtained for stress exposed samples (Figure S2). Analysis of dendrogram showed phenetic differences among stress treated fungal samples, suggesting how environmental cues change genetic makeup of an organism.

**Table 1:** Details of transposable elements (TEs) and oligonucleotides used in the study.

Class/subclass	Transposable element	Primer	Primer sequence (5′ - 3′)	References
LTR-retrotransposon	Grasshopper	GrhL2	AGGAGGAGGGATGGGCAAGA	This work
	MAGGY	MGYF0303-1	GGTGTCTTTGTAGGTGTTCG-ATCAGTTCC	42
	MGLR3	MGLRR1	GCTTAACCACTGCGCCATTC	This work
	MINE	MNR2	AGAGAGTTGTCCAAAGGCGT	This work
	Pyret	PyR1	CCCTTGTCCGTTTGAGATCA	This work
SINE like element	Mg-SINE	MGSI1	CGAGCCCGGCGTTAAATAAT	This work
DNA Transposon	Pot2	Pot2L2	TGAACCGGAGAAGCGTGAAA	This work
	Pot3	Pot3L2	CAGGAGGATGCAGAAATGTC	This work

**Figure 2:**
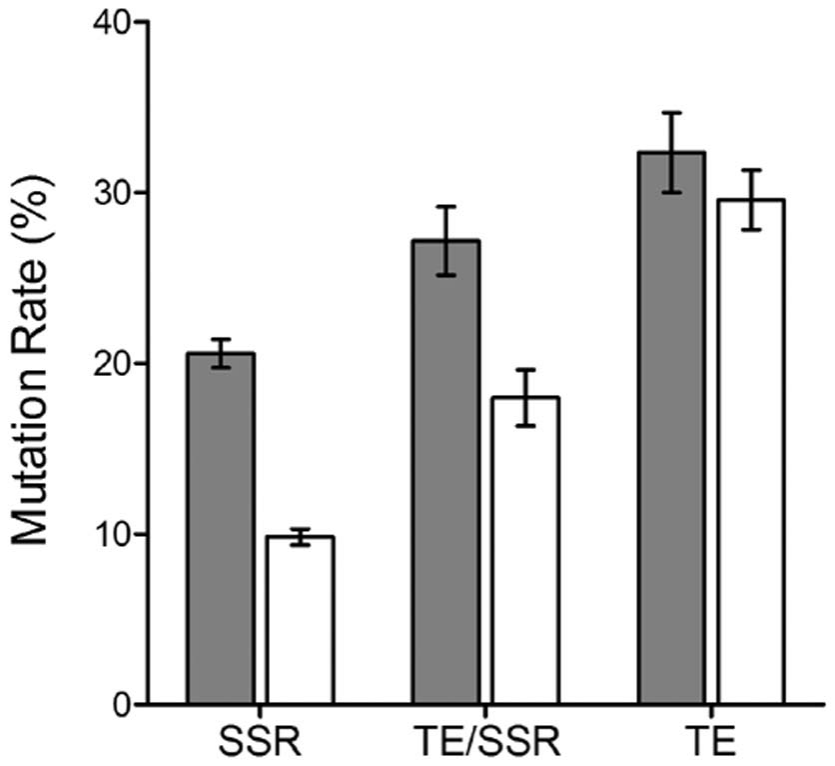
Stress induced genetic variations based on repetitive DNA elements. Mutation rates for transposable elements (TEs) were compared with simple sequence repeats (SSR) and TE/SSR regions. Mutation rates were calculated based on number of altered DNA bands generated in stress exposed samples as compared to control. Gray and white bars represent mutation rates determined for heat shock and copper stress exposed samples respectively. All the dataset are analyzed with two-way analysis of variance (ANOVA) with Bonferroni post-tests. Results were found extremely significant at p<0.0001. Data are presented as means ± standard error mean (SEM).

Since in *M. oryzae*, most of the mobile elements are present in the close proximity of repetitive DNA [14]; we used outward TE primer in combination with a primer designed for annealing at the 3′ end of a simple sequence repeat (SSR). This approach detects TEs inserted near SSRs and generates polymorphism based on both TE and SSR regions [16]. In addition to TE/SSR profiles, genetic alterations were also evaluated by single SSR primer with selective base(s) at 3′ or 5′ end. These primers generate DNA polymorphism based on inter simple sequence repeat regions [17]. Mutant bands generated in stress exposed samples were compared with untreated (control) samples and mutation rates obtained for TE, SSR and TE/SSR regions were compared (Figure 2). Two-way ANOVA analysis showed that repetitive DNA accounts for 64.6% of the total variance observed, whereas stress accounts 22.3% of the total variance (Table S3). Both the results were found extremely significant at p<0.0001. Among repetitive DNA, higher mutation rates were observed for TEs as compared to SSR and TE/SSR regions in copper and temperature stress exposed samples. The results showed greater contribution of TEs in stress induced DNA variations as compared to SSR regions.

### Role of TEs in genomic instability and genetic diversity of *M. oryzae*

**Figure 3:**
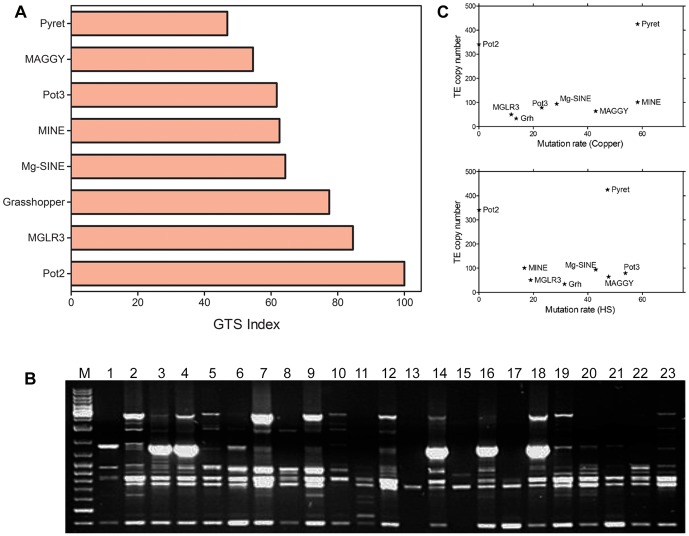
Stress induces genomic instability and genetic diversity in *M. oryzae*. A) Genomic template stability (GTS) index of *M. oryzae* transposable elements upon stress exposure. GTS index was calculated by analyzing mutant bands generated in stress exposed samples as compared to control sample. Data represents the means for stress exposed samples and was analyzed with one-way analysis of variance (ANOVA) with Dunnett's multiple comparison test (each group compared to the control). Data are presented as means of GTS index of stress exposed samples. Results showed Pyret with lowest GTS index, suggesting Pyret as the most unstable transposable element (TE) upon stress induction. B) High genetic diversity was observed among *M. oryzae* isolates. Genotypic profiles were obtained from 23 *M. oryzae* isolates from diverse geographical regions of India and Japan using PyR1 outward primer (Table 1) derived from LTR-retrotransposon Pyret. Results suggest bursts of Pyret under field conditions. C) Correlation between TE copy number (Y-axis) and mutation rate (X-axis) was determined. Pot2 with high copy number showed no genetic variations upon stress induction, whereas for Pyret linear correlation was observed between its copy number and mutation rate.

Among transposable elements, highest mutation rate was observed when genomic regions were amplified using PyR1 primer. Genomic template stability (GTS) index was calculated for TEs upon stress induction. GTS indices for copper and heat shock exposed samples were compared with control (untreated) sample using one way ANOVA followed by Dunnett's multiple comparison test. Both copper and heat shock exposed samples differed significantly from control sample for TE derived genetic variations at p<0.05. Two-way ANOVA on GTS data followed by Bonferroni post-tests showed that interaction between stress and TEs for the observed genomic variations is extremely significant at p<0.0001 (Table S4). The results showed LTR-retrotransposons Pyret and MAGGY with the lowest GTS of 47.2 and 54.7 respectively, followed by Pot3, MINE, Mg-SINE, Grasshopper, MGLR3 and Pot2 (Figure 3A). These findings suggested Pyret and MAGGY as major contributors to genomic instability in *M. oryzae* upon stress exposition. Since Pyret based primer PyR1 yielded highest number of mutated bands in stress exposed samples, it can be regarded as stress indicative primer.

For stress induced genomic modifications to have evolutionary importance, they have to be in sufficient number among fungal populations. LTR-retrotransposon Pyret as the most unstable TE was evaluated to examine genetic variability among geographically diverse isolates of *M. oryzae* from India and Japan (Table 2). Genotyping characterization of the selected 23 *M. oryzae* isolates led to the recognition of distinguishable and highly polymorphic genomic patterns (Figure 3B). These results showed potential application of Pyret based primer PyR1 for population studies among *M. oryzae* isolates.

**Table 2:** *Magnaporthe oryzae* isolates used for genetic variability studies.

S. No.	Isolate Name	State	Country
1	Almora	Uttarakhand	India
2	Malan	Himachal Pradesh	India
3	Gujarat	Gujarat	India
4	Nawagam-P203	Gujarat	India
5	Jagdalpur	Chhattisgarh	India
6	Karjat-cv4	Maharashtra	India
7	v-Karjat 2	Maharashtra	India
8	LVN 8.3	Maharashtra	India
9	KVL 7.3	Maharashtra	India
10	Mandya B11	Karnataka	India
11	Mandya NB11	Karnataka	India
12	KN 4.6.3	Kerala	India
13	KN 1.5.1	Kerala	India
14	Pondicherry	Pondicherry	India
15	Warangal	Andhra Pradesh	India
16	Nellore	Andhra Pradesh	India
17	Maruteru	Andhra Pradesh	India
18	Chiplima	Odisha	India
19	Titabar	Assam	India
20	Bankura	West Bengal	India
21	Ina 72	-	Japan
22	Ina 168	-	Japan
23	Ken 54-20	-	Japan

Since TE dynamics have often been associated with TE copy number, we tested possibility of correlation between TE copy number and transposition (Figure 3C). Here, we used mutation rate as an estimate of transposition rate by assuming that insertional events are correlated with the other mutational events and secondly that deletions are rare as compared to insertional events. TE copy number from *M. oryzae* isolate P131 [22] was used for correlation studies. The results showed a positive linear correlation between TE copy number and transposition rate of LTR-retrotransposon Pyret and MGLR3, whereas for other TEs no correlation was observed between TE dynamics and copy number. In spite of low copy number, high mutation rate for MAGGY was observed upon stress induction. On contrary, Pot2 with high copy number in the genome didn't show stress induced genetic changes.

**Figure 4:**
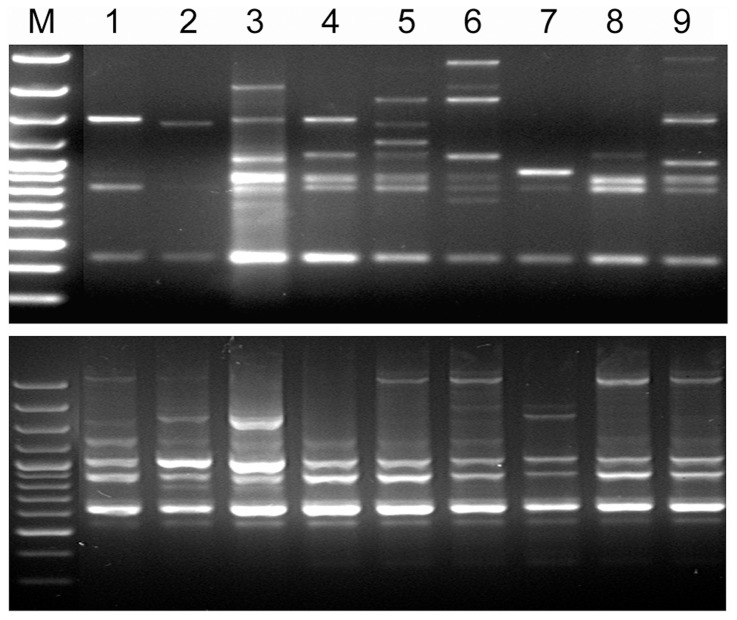
Genetic variability among *M. oryzae* isolates. DNA profiles were obtained for *M. oryzae* isolates using outward primers PyR1 (upper) and Pot2L2 (lower) derived from LTR-retrotransposon Pyret and DNA transposon Pot2 respectively. Details of PyR1 and Pot2L2 primers are provided in table 1. Lanes 1 to 9 represents *M. oryzae* isolates from similar agro-climatic region of India (Table 3). Lane M represents Fermentas GeneRuler 100 bp Plus DNA Ladder. As compared to Pot2, Pyret generated high intra-regional genetic variations among *M. oryzae* isolate.

To further validate our results, effects of agroclimatic conditions on genomic variability were determined by employing 9 isolates from Kerala, southern state of India (Table 3). Based on the stress induced genetic variations data, two representative TEs of *M. oryzae* including most stable (Pot2) and most unstable (Pyret) elements were selected. For LTR-retrotransposon Pyret, *M. oryzae* isolates from Kerala showed enormous genetic variability as compared to Pot2 (Figure 4). Results suggested that under particular agro-climatic conditions, genomic rearrangements based on Pyret are more common as compared to Pot2 in *M. oryzae* isolates. These findings further provided evidences of environmental effects on genetic makeup of a fungal pathogen.

**Table 3:** *Magnaporthe oryzae* isolates used for intra-regional genetic variability studies.

S. No.	Isolate Name	State
1	KN1.6.1	Kerala
2	KN1.9.3	Kerala
3	KN4.6.3	Kerala
4	KN1.4.1	Kerala
5	KN1.3.2	Kerala
6	KN 1.4.4.5	Kerala
7	KN1.3.2.2	Kerala
8	KN1.2.1	Kerala
9	KN1.5.3	Kerala

Further to confirm that the changes in banding patterns upon stress induction are result of TE transposition, we performed reverse transcriptase PCR for TEs MINE and Pyret (Figure S3). RT-PCR of MINE resulted in multiple transcript bands of WEIRD sequence of MINE retrotransposon [21] in samples exposed to copper and temperature stress. MINE transcript bands didn't amplify in control (untreated) sample. Similarly, induced expression of 638 bp long Pyret fragment was detected in stress exposed samples as compared to control (untreated) sample. Our results showed that both MINE and Pyret elements are expressed upon stress exposure, providing support to our theory of stress induced TE mediated genomic rearrangements in *M. oryzae*.

#### Heat shock and copper stress induces differential genomic instability pattern

**Figure 5:**
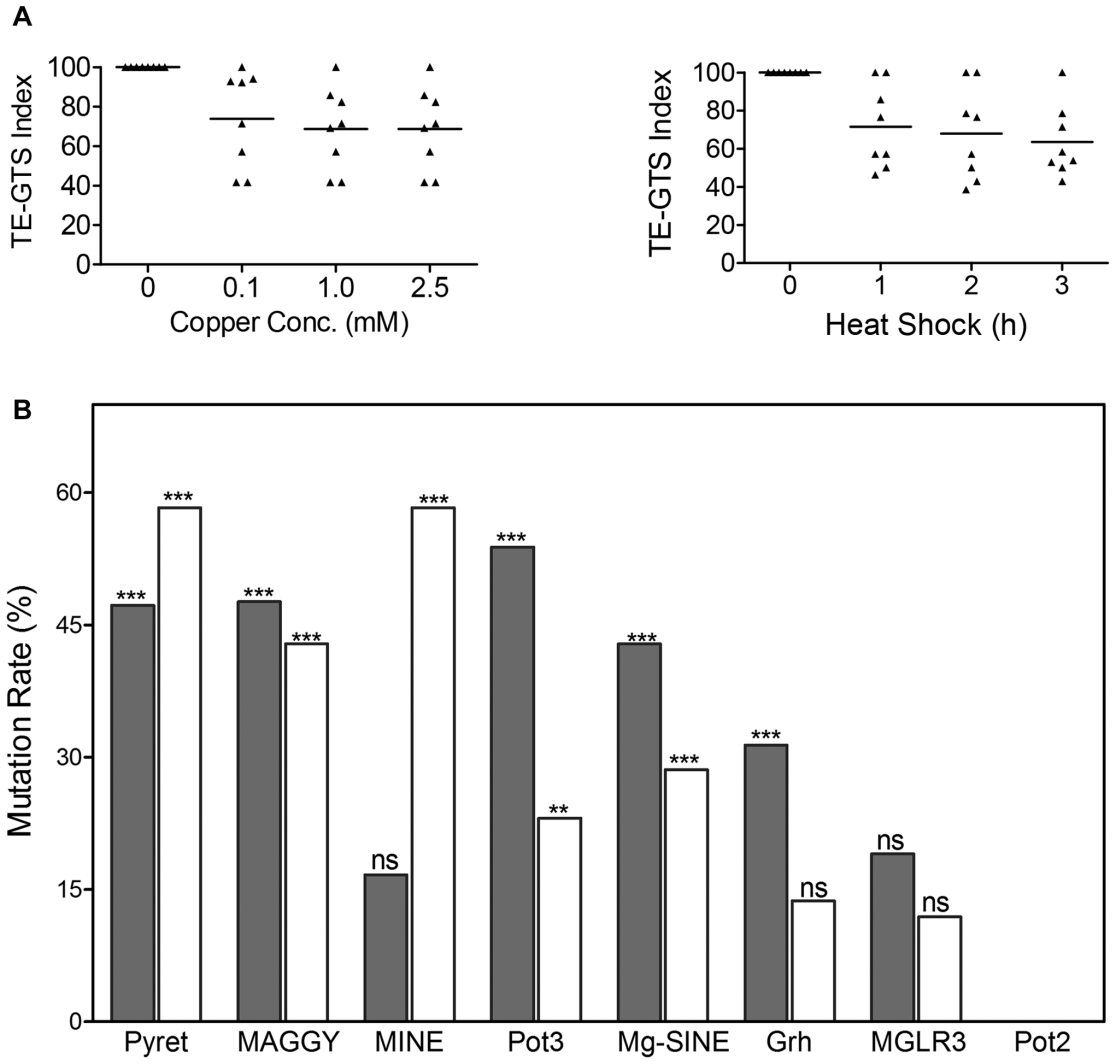
Differential effect of stress types on mutation rates of transposable elements. A) Genomic DNA templates from *M. oryzae* fungal cultures exposed to regime of durations and doses of heat shock (HS) and copper (Cu) respectively were analyzed for induced genetic variations. GTS index was determined for different doses of copper stress (left panel) and durations of heat shock (right panel). Data sets are significant at p<0.001 compared to control as analysed by two-way ANOVA with Bonferroni post-tests. B) Mutation rate data for different TEs upon exposure to copper (white) and heat shock (gray). DNA transposon Pot3 showed high mutation rates for heat shock samples. For copper stress, highest mutation rate was observed for LTR-retrotransposon Pyret and MINE. Data sets are the means of mutation rate. Data sets are analysed by two-way ANOVA with Bonferroni post-tests. ***denotes extremely significant results, ** highly significant results at p<0.001, ns non- significant at p<0.001.

To investigate correlation between induced genomic instability and types of stress conditions, *M. oryzae* cultures were exposed to different stress types, durations and doses. Differential genomic stability patterns were observed in responses to tested doses and durations of copper and heat shock respectively (Figure 5A). Average GTS index based on all TEs was slightly higher for copper stress (70.4) compared to heat shock (67.7) suggesting higher genomic rearrangements upon exposure to heat shock as compared to copper stress. The observed genetic variations were found specific to stressors and a positive correlation was determined between mutant bands generated for TEs and dose/duration of stress (r = 0.65 for copper and 0.91 for heat shock). Fungal Cu (1.0 mM and 2.5 mM) and heat shock (3 h) samples displayed highest genetic variations. For heat shock, the lowest genomic stability was observed for Pot3 followed by MAGGY, Pyret, Mg-SINE, Grasshopper, MGLR3 and MINE; whereas in copper exposed samples, the lowest genomic stability was found for Pyret followed by MINE, MAGGY, Mg-SINE, Pot3, Grasshopper and MGLR3 (Figure 5B).

**Figure 6:**
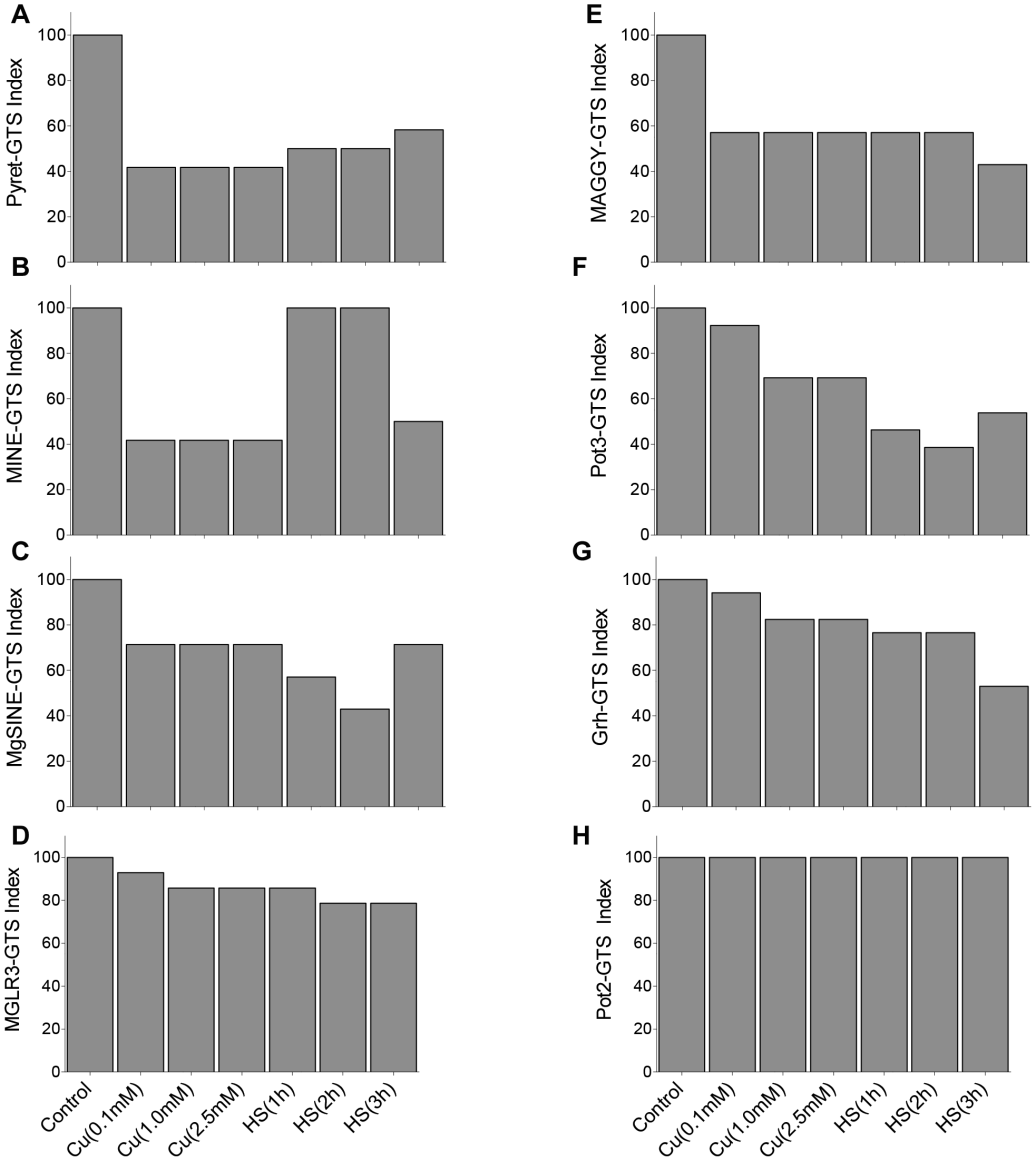
Genomic template stability (GTS) index of *M. oryzae* transposable elements upon stress exposure. GTS index of transposable elements were calculated based on the number of mutant bands obtained in *M. oryzae* samples exposed to regime of heat shock (HS 1-3 h) and copper (Cu 0.1, 1.0 and 2.0 mM) stress. For each TE, GTS index (Y-axis) for *M. oryzae* stress exposed samples (X-axis) is determined. Data are presented as means from 3 independent experiments. Panels A to H represents GTS index of TEs Pyret, MINE, Mg-SINE, MGLR3, MAGGY, Pot3, Grasshopper (Grh) and Pot2 respectively. Data sets are significant compared to control as analysed by two-way ANOVA with Bonferroni multiple comparison post-tests. Overall, LTR-retrotransposons Pyret (A) and MAGGY (E) showed lowest genomic stability upon stress induction. Pyret showed slightly lower GTS for copper concentrations tested, as compared to heat shock (A). For MAGGY, GTS was not affected by stress type/dose/duration except for HS-3h sample (E). For copper stress, LTR-retrotransposon MINE showed lowest GTS (B). LTR-retrotransposons MGLR3 (D) and Grasshopper (G) showed high GTS index (76 and above) for all stress exposed samples except for HS-3h (52.9). Similarly for all copper doses, Mg-SINE showed similar GTS index (71.4 and above), whereas low GTS of 57.1 and 42.9 for HS-1h and HS-2h samples respectively (C). DNA transposon Pot3 (F) showed more sensitivity towards heat shock, whereas Pot2 (H) showed 100% genomic stability upon stress exposition. Results suggested genome template stability differs for type/family of TEs upon stress exposition.

For each transposable element, effects of stress dose/duration on GTS indices were investigated (Figure 6). For this analysis, mean values of GTS index data from three independent experiments were used. Data sets were found significant compared to control as analysed by two-way ANOVA with Bonferroni multiple comparison post-tests. Overall, LTR-retrotransposons Pyret (Figure 6A) and MAGGY (Figure 6E) showed lowest genomic stability upon stress induction. Pyret showed slightly lower GTS for copper samples as compared to heat shock. For MAGGY, GTS was not affected by stress type/dose/duration except for HS-3h sample. Pyret derived primer PyR1 generated marker specific to copper stress (Figure S1). Such DNA markers present in the exposed samples can be regarded as stress diagnostic DNA fragments. For all copper doses, Mg-SINE showed 71.4 GTS index, however, HS-1h and HS-2h samples resulted in GTS index of 57.1 and 42.9 respectively (Figure 6C). Similarly DNA transposon Pot3 was also found more sensitive to heat shock with low GTS index (46.2) as compared to copper stress (76.9) (Figure 6F). On contrary, LTR-retrotransposon MINE showed lowest GTS (41.7) for copper stress exposed samples, whereas higher GTS index (83.3) was observed upon exposure to thermal stress (Figure 6B). These findings suggest MINE is more susceptible to copper stress to cause induced DNA variations. Among different heat shock samples, MINE showed 100% genomic template stability for HS-1h and HS-2h samples. However, exposure to longer duration (3 h) of thermal stress decreased MINE-GTS index by almost 50%, suggesting effects of stress duration on induced genomic rearrangements based on MINE. Another LTR-retrotransposon MGLR3 showed high GTS indices (76 and above) for all stress exposed samples (Figure 6D). DNA transposon Pot2 showed 100% genomic template stability upon stress exposition (Figure 6H). Interestingly, results based on differential GTS indices for TEs provide evidences where stress induced changes in genome are related to the type and family of transposable elements.

#### LTR-retrotransposons MAGGY, Pyret and MINE sequences in the upstream region of pseudogenes

To examine where these DNA alterations occur in the genome, whether genomic repeats alters similar genomic sites under stress conditions or are there any stress sensitive genomic spots, we cloned and sequenced representative stress induced mutant bands. These bands were selected based on clarity, frequent alterations among samples and reproducibility among biological and experimental replicates. Each of these DNA sequences represented altered or unstable loci of *M. oryzae* genome upon stress exposure. The sequences of selected altered DNA bands were located on *M. oryzae*'*s* genome by performing homology searches. Analysis led to the identification of the genomic regions from where mutant bands were derived. We referred these regions as stress inducible regions or unstable loci of the genome. Analysis led to the identification of heat shock and copper stress sensitive genomic regions in *Magnaporthe* and revealed that induced genetic alterations occurred in genomic regions of chromosomes 2, 3, 5 and 6 (Table 4).

**Table 4:** Summary of the cloning and sequence analyses of representative stress altered DNA bands.

Mutant Band (Primer/Amplicon size in bp)	Repetitive DNA target	Stress condition	Chromosome location of gene/fragment	Sequence homology to genomic region	Details of homologous genomic region/gene
Pot3L2/1150-1	Pot3	Control and heat shock	Chr 2:2902978-2905478	Exon and downstream of MGG_01268	Nuclear localization sequence binding protein with conserved RNA recognition motif (RRM)
Pot3L2/1120	Pot3	Control and absent in stress samples	Chr 2:2902978-2905478	Exon and downstream of MGG_01268	Nuclear localization sequence binding protein with conserved RNA recognition motif (RRM)
Pot3L2/1150-2	Pot3	Copper (0.1 mM)	Chr 2:731973-736317	Exon of MGG_15537	Putative chromatin remodeling protein with conserved copper binding domain
PyR1/780	Pyret	Heat shock (2 h)	Chr 6:3884060-3885199	Upstream of MGG_09851	Pseudogene with premature stop codon
(AG)_8_G/MGYF0303-1/900A	MAGGY/(AG)_8_G	Copper (1.0 mM)	Chr 3:292697-293508	Upstream of MGG_16600	Pseudogene with premature stop codon
(AG)_8_G/MGYF0303-1/900B	MAGGY/(AG)_8_G	Copper (2.5 mM)	Chr 3:292697-293508	Upstream of MGG_16600	Pseudogene with premature stop codon
(GA)_8_T/MNR2/1200	MINE/(GA)_8_T	Heat shock (2 h)	Chr 5:3158315-3156983	Upstream and coding region of MGG_17470	Pseudogene with premature stop codon

Representative stress altered DNA bands generated from LTR-retrotransposons Pyret, MAGGY and MINE were analyzed in detail. Altered band of 780 bp generated from heat exposed (2 h) sample using Pyret derived primer PyR1 was sequenced. BLAST search against *M. oryzae* database showed fragment PyR1/780 was located to 349 bases upstream of the locus MGG_09851, a pseudogene with premature stop codons and missing 5′ and 3′ ends. For LTR-retrotransposons, we also sequenced altered DNA bands generated using SSR/TE based primers. For MAGGY, 900 bp long altered band was amplified using primers (AG)_8_G/MGYF0303-1 only in copper and control samples and was absent in heat shock treated samples. We sequenced 900 bp fragment (AG)_8_G/MGYF0303-1 from samples exposed to two different copper doses (1.0 and 2.5 mM) with an aim to check whether co-migratory altered bands were derived from similar region. The alignment of DNA sequences of 900 bp band from Cu (1.0 mM) and Cu (2.5 mM) samples showed these two co-migratory bands as distinct fragments. BLAST search against *M. oryzae* database showed that these bands are derived from different genomic regions with the major portion showing homology to the upstream region of locus MGG_16600 (812 nt), apparently a pseudogene with premature stop codons. For LTR-retrotransposon MINE, 1200 bp long altered band (GA)_8_T/MNR2/1200 from heat exposed (2 h) sample was sequenced (Table 4). Interestingly, (GA)_8_T/MNR2/1200 was located to chromosome 5, showed an overlap of sequenced fragment with the upstream and coding regions of gene MGG_17470, apparently a pseudogene with premature stop codons. The presence of MINE primer binding site within the MGG_17470 gene suggests interruption of coding region of the gene by MINE element. These findings illustrated the presence of LTR-retrotransposons Pyret, MAGGY and MINE sequences within upstream regions of pseudogenes.

#### Pot3 transposon sequences in coding regions of NLS-binding and chromatin remodeling proteins

Pot3 primer amplified stress induced band of 1150 bp from control and Cu 0.1 mM samples respectively (Table 4). This band didn't amplify from other stress exposed samples. To find out whether, these two fragments are derived from the same or different loci; we aligned DNA sequence of fragment amplified from control and Cu 0.1 mM samples followed by homology searches. Analyses showed these two sequences didn't show any homology with each other, which means these co-migratory bands represent different regions of the *Magnaporthe* genome. Detailed analysis of amplified fragment Pot3L2/1150-1 showed homology to exon3 of locus MGG_01268 and its downstream region. Analysis of mutant band Pot3L2/1120 showed amplification of 1120 bp band only in control sample and disappearance in all stress exposed samples. This fragment was also located to locus MGG_01268 similar to the fragment Pot3L2/1150-1. Locus MGG_01268 was predicted to encode putative nuclear localization sequence binding protein of 486 amino acids (2501 nt) with two RNA recognition motifs (RRM). RRM motifs are flanked by region of low complexity and are probably diagnostic of a RNA binding protein with implications in regulation of alternative splicing. Interestingly, yeast ortholog of MGG_01268 is *NSR1*, nucleolar protein which binds nuclear localization sequences [28], plays a role in fitness [29] and heat sensitivity [30].

Another fragment amplified using Pot3 primers (Pot3L2/1150-2) from Cu 0.1 mM sample was located within coding region of the locus MGG_15537, a hypothetical protein. Analysis of amino acid sequence for protein encoded by locus MGG_15537 showed presence of conserved tyrosinase copper-binding domain signature which binds two copper ions (CuA and CuB). Further analysis showed presence of conserved PWWP domain and a putative chromatin binding site in the MGG_15537 encoded protein. The PWWP domain has a conserved Pro-Trp-Trp-Pro motif and is present in proteins of nuclear origin. PWWP motif has been suggested to involve in protein-protein interactions, chromatin remodeling and transcriptional regulation [31]–[33]. The function of this domain is still not known precisely. Recently, proteins containing PWWP domain such as EXPAND1/MUM1 have been shown to play role as an architectural component of the chromatin, promoting cell survival under stress condition [34]. Our findings of DNA changes due to insertion of Pot3 sequences exclusively in copper exposed samples suggest role of MGG_15537 encoded protein containing tyrosinase copper-binding domain signature and PWWP domains in maintaining genome stability under copper stress conditions. Interestingly, both the stress specific loci identified in our study (MGG_01268 and MGG_15537) showed the presence of Pot3 transposase sequence. However, upon homology search with *M. oryzae* genome sequence database, Pot3 mutant band sequences were found absent in these loci, suggesting the sequenced mutated bands are generated due to changes in the genome after exposure to copper stress.

## Discussion

The organisms diversify and evolve their genome to survive under unfavorable environments [35]. For our study, a model plant pathogenic fungus *M. oryzae* was employed. This pathogenic fungus easily adapts when introduced into new geographical areas and surroundings. On contrary to mainstream assumptions, in *M. oryzae* expression of putative heat shock or chaperone related genes remained unaltered upon exposure to thermal stress [36]. *M. oryzae* genome evolution model was proposed with a history of episodic transposable element amplification and intrusion, responsible for shaping the pathogen's genome [37]. This study was designed to determine the effects of field stress at DNA level in *M. oryzae*. Here, we investigated how unfavorable conditions due to heat shock and copper stress influence genome stability, what kind of genomic components are involved and whether genome stability is dependent on dose/duration of stress. To address these questions, PCR-based approach was used where TE based variations were evaluated upon stress exposure. Significant TE based genomic instability was observed in stress exposed samples when compared to positive control. This is the first study where *M. oryzae* TEs are compared for genomic stability upon stress exposition and LTR-retrotransposon Pyret being identified as the most unstable TE with a potential for population studies. Our results based on genomic stability data showed both copper and temperature stress induces TE mediated genomic rearrangements in *M. oryzae*, suggesting that the higher copper content of soil and temperature stress are among the important environmental factors responsible for the high genetic diversity of this pathogen under field conditions.

**Figure 7:**
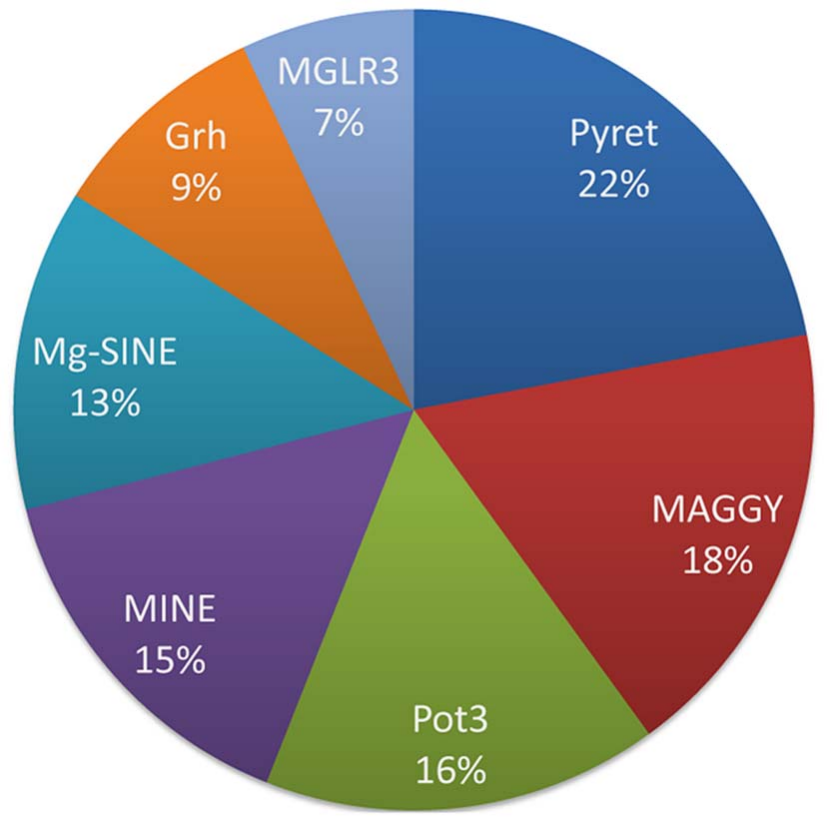
Relative contributions of the transposable elements (TEs) in stress induced DNA variations in *M*. oryzae. Pie chart depicts estimated relative contributions of the transposable elements (TEs) attributable to stress induced DNA variations in *M*. *oryzae* genome. The listed TEs include members of class I (MAGGY, Pyret, Grasshopper, MGLR3, MINE and Mg-SINE) and II (Pot3 and Pot2) families. Estimate is based on the mutation rate data calculated using mutant bands generated in stress exposed sample as compared to control. LTR-retrotransposons Pyret and MAGGY accounted for 40% of the observed genetic variations upon stress induction. The remaining 60% variations are attributed to Pot3, MINE, Mg-SINE, Grh and MGLR3.

Induced genomic instability observed for *M. oryzae* was attributed to Pyret and MAGGY followed by Pot3, MINE, Mg-SINE Grasshopper and MGLR3 (Figure 7). The observed stress induced DNA variations are reflected by profiles based on different transposable elements and included modifications of band intensity, appearance of new bands and disappearance of control bands. Such variations could represent a large proportion of overall mutations and may affect genome structure and gene functions leading to evolutionary consequences. Observed DNA variations could be the result of changes in oligonucleotide priming sites due to genomic rearrangements, point mutations, DNA damage and activation of transposable elements. Mutations, large deletions or homologous recombination can cause the structural changes or changes in DNA sequences. Such genomic changes might be responsible for the accessibility of oligonucleotide priming sites, resulting in appearance of new bands in stress exposed samples [38].

In *M. oryzae*, differential response observed for TE based variability to regime of stress conditions suggests its cellular capacity to sense and discriminate among stressors at DNA level, affecting genomic charge and architecture at variable levels. This response might be a reflection of strategies employed by fungal cells to cope with the ever-changing environment. Since stress reduces growth or performance, presence of appropriate genetic variability is expected to lead to evolutionary changes in populations where a consistent stress is occurring [39]. There are now considerable evidences for such evolution, producing constitutive adaptations of an organism in response to stress, which are specific to the stress concerned.

Although our data show environmental cues contribute to induced genomic variations in *Magnaporthe*, for stress induced genomic changes to have evolutionary importance, they have to be in sufficient number among fungal populations. Therefore, results were validated by performing genetic variation studies among *M. oryzae* isolates for representative TEs with low and high GTS indices upon stress induction. As compared to Pot2, higher genetic variations observed for Pyret provide further evidences of environmental effects on genetic diversity of a fungal pathogen. Further research, in particular a demonstration that stress-induced transpositional events can be transmitted to a progeny in natural selection, will be necessary. In addition, isolates within a population display considerable heterogeneity in their responses to stress, therefore, further investigation is required to study the impact of these stressors on TEs identified with low GTS at population and subpopulation levels as well as for their presence in vicinity of genes involved in pathogenicity and stress signaling network.

Under stress conditions, pathogen's survival depends on its ability to develop tolerance, resistance or avoidance mechanisms. We obtained copper tolerant *M. oryzae* mutants (Figure 1A) and also found accumulation of copper within *Magnaporthe* cells. The accumulation and high tolerance of copper by M. oryzae cells could be perhaps correlated with the continuous usage of copper-based fungicides in rice fields to eradicate the blast fungus. We observed that genetic changes were phenotypically neutral up to 1.0 mM Cu concentration, whereas at higher copper concentrations (2.5 mM), the variable phenotype was also observed along with genetic variations. These results suggest that the accumulated stress induced novel genetic variations might be phenotypically neutral under a normal range of environments. However, under adverse environmental conditions, these variations can be adaptive to facilitate the pathogen's survival. Our results corroborates with simulation studies which noted that a population showing 10-fold stress-inducible mutator phenotype will adapt up to 15% faster than a non-mutator population, whereas a population having a 100-fold stress-inducible mutator phenotype will adapt up to 38% faster [40].

TEs with variable GTS index for stress dose/duration suggested that genetic alterations are dependent on the type of DNA targets as well as on the stress conditions. Low GTS indices of Pyret and MAGGY indicate high activity of these elements in *M. oryzae* upon stress exposition. This is the first report illustrating LTR-retrotransposon Pyret contributions in the stress induced genomic changes as well as its application in genetic diversity studies. Low genomic stability of MAGGY observed in this study corroborates with earlier findings which showed stress induced promoter activation and transpositions of LTR-retrotransposon MAGGY [41] and contribution of MAGGY in genetic diversity of *M. oryzae* [17], [42]. Nevertheless, another transposon Pot3 also showed low genomic stability index (31). Our results showed potential of Pyret, MAGGY and Pot3 as genomic targets for population biology and evolutionary studies. Our findings showed TEs MINE, Mg-SINE, MGLR3 and Grasshopper are also active in blast pathogen, though relatively less functional. These results support previous speculations of Mg-SINE [43] and Grasshopper [44] activity in *M. oryzae*.

Stress induced burst of TEs in fungal pathogen might lead to the rapid genome diversification by causing aberrant changes in genome including gene duplication and exon shuffling, processes responsible for gene evolution. In *M. oryzae*, retroelements represent 5.4% of the genome [14] and show limited but sporadic distributions [42], speculated due to recent invasion of these elements in *Magnaporthe* genome through horizontal gene transfer. The rapid expansion of these elements in the genome provided *M. oryzae* with an evolutionary advantage and is likely to be temporally related to the evolution of pathogenicity or a host shift by the fungus. Based on the observation of high stress induced genomic instability of Pyret and MAGGY, we proposed an alternate explanation that stress could be a major factor, if not the only one, for the sporadic expansion of retrotransposons in *M. oryzae*. Based on our and prior studies [45], it could conceivably be hypothesised that the contribution of transposon amplification in genome expansion is much higher than anticipated by the evolutionary model.

Low correlation observed between TE copy number and mutation rate might be due to the variable selection coefficients, deletion rate and history of *M. oryzae* TEs. For Pyret, a positive correlation was observed between mutation rate and copy number in *M. oryzae* suggesting this element is ancient and still active in *M. oryzae*. However, LTR-retrotransposon MAGGY with low copy number showed high mutation rate suggesting MAGGY as relatively new and active element in *M. oryzae*, supporting previous reports [41]–[42]. MAGGY carriers occurring in the natural population of *M. oryzae* possess 35–40 copies of MAGGY [46]. But when MAGGY element was introduced into a naive genome of *M. oryzae*, its copy number in transformants (20–30 copies) couldn't attain the copy number as observed in the natural evolution of *M. oryzae* and MAGGY [24]. This discrepancy could be explained by our results where MAGGY is shown to be sensitive to stress, suggesting MAGGY activity in natural population is induced by environmental cues. On contrary to MAGGY, despite the high copy number, Pot2 didn't show any changes upon stress induction. This observation may be the result of the history of Pot2 within M1477 isolate. Pot2 could have been active in this isolate a long time ago, had even invaded the isolate and then stopped being active [42].

TE insertions can sometimes have beneficial effects and several putative cases of site preferences and adaptive insertions have been detected. TEs are able to change the genetic environment of the locus into which they insert, therefore, the most important point for the impact of induced changes is the locations of insertions. The reasons why insertions are beneficial have been speculated but still not been fully understood. Prior studies showed enhanced transposition of MAGGY during mating and under abiotic stress [41]–[42], novel insertions were not characterized. Sequencing of amplified mutant bands using TE and/or TE/SSR primers led to the identification of genomic regions which showed signs of stress susceptibilities. While the cloned sequences represent members of repetitive DNA elements, we asked whether the detected altered genomic profile is random or specific or have preferred sites for rearrangements. How does it differ for two different types of stressors and different transposable families? To address this question, more detailed analyses was performed using seven selected altered DNA fragments for their genomic locations and their nearby genes. Interestingly, we found that stress susceptible spots differed for LTR-retrotransposons and DNA transposons insertions. Upon stress induction, retrotransposons MAGGY, Pyret and MINE sequences were identified within upstream regions of pseudogenes. These results add to the evidence that pseudogenes have arisen through the retro-transcription of mRNAs and are located exclusively in the same heterochromatic regions as transposable elements [47]. Prior studies have noted DNA transposon Pot3 sequences in the upstream and coding regions of avirulence genes leading to the changes in the virulence spectrum of this fungal pathogen [18]–[19]. We found Pot3 sequences within coding regions of two new loci encoding putative chromatin remodeling and NLS-binding proteins (Table 4). Pot3 presence within chromatin remodeling protein indicates its role in epigenetic phenomena under stress conditions. However, the mechanism by which Pot3 elements target these sites requires elucidation. Our results provide support for the prior findings of the role of epigenetic changes in stress induced activation of TEs [48]–[49]. In terms of evolution, this is probably one of the crucial key points [8]; to date little has been published in this field. Therefore, it is imperative to carry out theoretical and experimental investigations to define the impact of TE induced epigenetic phenomena at population level.

Dynamic response to changing conditions in the environment is an essential property of all biological systems. Whereas, extensive research over the last several decades has elucidated numerous molecular responses to environmental stress, it is much less known how these translate into organismal-level responses. Our results suggested that under adverse environmental conditions, TEs can be interpreted as stress adaptors of cells, aiding to fungal fitness. The high number of stress induced variations and amplifications of TE sequence derived altered fragments from upstream, downstream and coding regions of genes as well as psuedogenes observed in this study reflect the fundamental mechanisms of fungal genome evolution.

**Figure 8:**
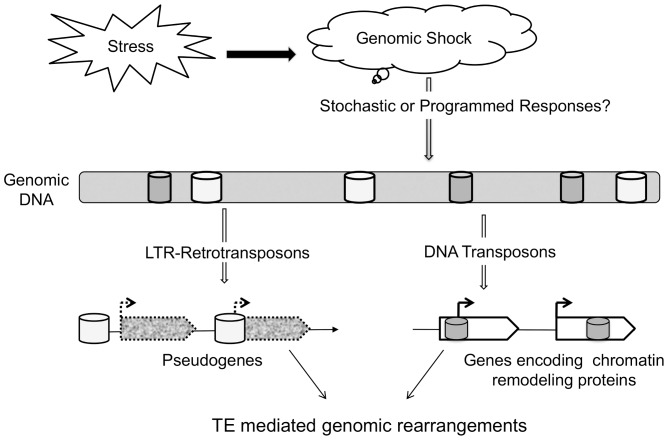
Model for TEs role as stress capacitors to promote genomic rearrangements in fungal pathogen. In the presence of appropriate stress, cells experience genomic shock that generates signals (stochastic or programmed) to induce transpositional activity. This leads to the insertion of transposable elements within regulatory and coding regions of genes resulting in genomic rearrangements.

“The First Rule of Adaptive Evolution”: Break or blunt any functional coded element whose loss would yield a net fitness gains [50]. We present a model to show how stress generated genomic shock leads to TE mediated genomic rearrangements (Figure 8). It is not clear whether signals for TE activity are stochastic or programmed. Genomic repeats, and in particular LTR-retrotransposons, were found to be rich sources of genomic variations. Responses to stress - whether it is due to change in epigenetic frameworks or transcriptional control or pseudogene formation, can force cells to adapt to generate fitter genotypes [51]. The identification of stress sensitive spots of genomic variations has an implication for better understanding of various areas: such as gene regulation; genome evolution and speciation; and the idea of the environment influencing genome structure of this fungus. With this understanding, we are only beginning to glimpse the complexity of possible stress induced genome dynamics. Further work will be required to understand the interactions among stress, *M. oryzae* population and TE dynamics, and its consequences to pathogenic events and hosts.

## Materials and Methods

### Microbial strains and growth conditions

*M. oryzae* isolate M-1477 was obtained from Microbial Type Culture Collection, Institute of Microbial Technology, India. Isolates listed in tables 2 and 3 were provided by Dr K. K. Muralidharan, Dr. S. S. Gnanamanickam and Dr. Y. Tosa. The cultures were maintained in potato dextrose broth (PDB; HiMedia) and stored as −80°C glycerol stock. For routine use, fungal culture was maintained on potato dextrose agar (PDA, HiMedia) plates at 4°C. Cultures were routinely manipulated at 25°C. *Escherichia coli* XL-I-B competent cells were used as a host for plasmids and were grown in Luria-Bertani (LB) medium (Difco). Solid media were made with 1.8% Bacto agar (Difco).

### Copper uptake and growth rate

To measure prolonged effect of copper, *M. oryzae* cultures were grown on PDA plates in the absence or presence of copper (0–5 mM) at 25°C for 7 days. For this study, environmentally relevant copper concentrations were selected. To examine copper uptake and its effect on *M. oryzae* growth, cultures were grown in 100 ml of CM media (3% yeast extract, 5% sucrose and 3% casamino acid) at 25°C for 4 days with continuous shaking at 100 rpm. After 4 days of growth, cultures were exposed to copper (0–5 mM) for 18 h. The mycelia of the cultures were harvested by filtering through muslin cloth and washed extensively with distilled water to remove media. Fungal mass obtained after harvesting was divided into two halves to estimate Cu uptake and dry weight. To examine Cu accumulation by *M. oryzae* cells, the fungal samples were dried at 70°C for 6 h. Dried fungal mass was digested in an acid mixture of HNO3:HClO4 (5∶1 v/v) at 80°C. Copper content was estimated by GBC 932 B+ atomic-absorption spectrophotometer (GBC, Melbourne, Australia) using air–acetylene flame. To evaluate the effect of Cu on growth, fungal mass was dried in a vacuum oven at 80°C until constant weight. Data from at least 3 independent experiments were used to calculate inhibitory rate (IR, %), where IR = (*1−x/y*)×100, where x and y were the average growth rate of the treated and untreated (control) samples, respectively. Copper concentration values for fungal growth inhibition by 50% (IC50) were calculated from dose-response curves.

### DNA manipulations

Standard conditions for molecular cloning, transformation and electrophoresis were used (Sambrook 1989). All oligonucleotides based on DNA sequences of transposons and retrotransposons were synthesized by Metabion International AG (Germany). GeneRuler DNA markers and Fast-Link DNA ligation kit were purchased from Fermentas and Roche Diagnostics (Mannheim, Germany), respectively. Plasmid DNA was isolated from *E. coli* by the alkaline lysis method, employing a QIAprep spin miniprep kit from Qiagen. Purification of PCR mixes and DNA fragment isolation from agarose gels were performed by using QIAquick PCR purification and gel extraction kits (Qiagen).

### DNA extraction

*M. oryzae* isolates were inoculated on PDA plates at 25°C for 5 days. For DNA extraction, a 5 mm piece of agar with actively growing hyphae was inoculated in 100 ml of CM media and incubated at 25°C in a rotary shaker at 100 rpm. After 4 days of growth, cultures were exposed to regime of copper concentrations (0–2.5 mM) for 18 h and heat shock at 42°C (1–3 h). DNA samples from stress treated and untreated fungal cultures were isolated, purified and quantified [12].

### PCR Amplifications

DNA sequences of transposable elements were obtained from *M. oryzae* genome database. Primers were designed for 8 different transposons and retrotransposons sequences of *M. oryzae* using PRIMER software v1.01 (Table 1). All the primers designed were outward primers amplifying either left or right side sequences of transposable elements. PCR reactions and program were optimized to obtain clear, comparable and reproducible results. For TE based profiles, the genomic DNA samples were amplified in 25 μl of reaction mixture containing 50 ηg DNA, 1× *Taq* polymerase buffer (10 mM Tris-HCl pH 8.3, 50 mM KCl, 2.5 mM MgCl_2_, 0.01% gelatin), 150 μM of each dNTP, 0.4 μM TE primers (Table 1) and 0.5 U *Taq* DNA polymerase. ISSR profiles were generated following previously reported protocol [17]. To obtain TE profiles in combination with SSR repeats, 0.4 μM of TE primers were used in combination with 0.6 μM ISSR primers. The PCR program was optimized and thermal cycler (Eppendorf Netheler-Hinz, Hamburg, Germany) was programmed for 5 min denaturation at 94°C, followed by 40 cycles of 94°C-45 s, 55°C-45 s and 72°C-1 min followed by an additional elongation step at 72°C-5 min. The amplification products obtained were analyzed on 2.0% agarose gel in 1× Tris–borate–EDTA buffer (pH 8.3) at 100 V and stained with ethidium bromide (0.5 μg/ml). GeneRuler DNA markers were loaded in each gel (Fermentas). The bands were visualized by their fluorescence under ultraviolet illumination and documented using a gel documentation and image analysis system (Syngene, UK).

### Data scoring

All PCR analyses were performed several times to obtain stable and reproducible band profiles. The DNA profiles included in the study were based on at least two independent amplifications. A negative control (without DNA template) was included in all PCR reactions. Each amplification product identifiable after electrophoresis was considered as a DNA marker and scored across all samples. The presence and absence of each band was determined by making binary matrix (1 for band presence and 0 for absence) for each sample. A change of band intensities defined as an increase or decrease of the intensity of the signal of a band by ≥50% in the stress DNA by direct visualization when compared to normal DNA [52]. A change of band intensities was also scored across all samples as compared to control.

### Genomic template stability

Mutation Rate (MR) of each treatment group was calculated using altered DNA bands as number of altered bands/total number of bands in control x 100. To confirm the statistical significance of the data, results were analyzed by performing two-way analysis of variance (ANOVA). Genomic template stability (GTS) was calculated for each primer as 100−(100 a/n) where, a  =  number of mutated bands and n  =  number of bands in the control sample [53].

### Statistical analysis

Copper uptake data were fitted by non-linear regression using one phase exponential association equation *y* = *y*_max_ (1−e^−^ *kx*), where *y*_max_ is the maximum cellular accumulation of copper, *k* is the rate constant, and the initial rate of uptake is calculated as *k*·*y*_max_. For DNA variations, data obtained for the stress-exposed cultures and positive control was analyzed by one-way ANOVA. If ANOVA results were found significant (*P*<0.05), then Dunnett's multiple comparison test was applied. This comparison was performed to ensure that data sets for stress exposed samples were significantly different from the positive control. Pooled data for each experiment group are expressed as mean and SEM. Mutation rate and GTS index data were analysed using one-way ANOVA or two-way ANOVA with Dunnett's or Bonferroni multiple comparison post-tests. All statistical data were analyzed using the statistical and graphical functions of the GraphPad Prism 5.02 statistical software package (GraphPad Software, Inc., California, USA).

### Genetic similarity coefficient

The effect of various treatments and extent of genetic alterations was also assessed on the basis of the distance among samples. Numerical analysis based on DNA pattern obtained from stress treated samples was compared with untreated samples (control) via hierarchical cluster analysis. Simple matching similarity coefficients were calculated using the SIMQUAL program. Matrices of similarity coefficients were subjected to unweighted pair group method with arithmetic mean (UPGMA) to generate a dendrogram using NTSYS-pc (Numerical Taxonomy System, version 2.02).

### RT-PCR

Total RNA was extracted from *M. oryzae* mycelia grown in liquid cultures with RNeasy mini kit (Qiagen) according to the manufacturer's instructions. First-strand complementary DNA was synthesized using first strand cDNA synthesis kit (AMV) from Roche. The reaction was performed in a final volume of 20 μl consisting of 1× reaction buffer (10 mM Tris, 50 mM KCl_2_; pH 8.3), 5 mM MgCl_2_, 1 mM dNTP, 50 U RNase inhibitor, 1.6 μg oligo (dT)_15_ primer, 1 μg RNA and 16 U AMV reverse transcriptase (Roche). The mixture was incubated at 25°C for 10 min and then at 42°C for 60 min. RT enzyme was inactivated at 99°C for 5 min followed by incubation at 4°C for 5 min in a thermal cycler (Eppendorf Netheler-Hinz, Hamburg, Germany). First strand cDNA was amplified using high-fidelity enzyme Phusion HF (NEB, England). Amplifications were performed in a final reaction volume of 25 μl consisting of 1× Phusion HF buffer, 300 μM dNTPs, 0.4 μM primer, 3 μl of cDNA and 0.5 U Phusion DNA polymerase. DNA fragments of MINE were amplified using primer pairs WEIRD125-forward (5′-CCTAAGCACCGTCACTACAC-3′) and WEIRD967-reverse (5′-TTCGGATGGCGTAGGTGTTT-3′) [21] and a PCR fragment for Pyret was amplified using primers Py-FW1 (5′-GATTTACGAACGCCGACA-3′) and Py-RV1 (5′-GTCCTAATGTGGTGGGTT-3′) [54]. The PCR protocol involved an initial denaturation step at 98°C for 1 min, 37 cycles of 98°C-10 s, 55°C-30 s, and 72°C-1 min, followed by an additional elongation step at 72°C for 5 min. Control amplifications of a 200-bp fragment of the *Magnaporthe* actin gene was performed using primers Act5 (5′-TGGCACCACACCTTCTACAA-3′) and Act32 (5′-CGGAGTCGAGCACGATACCA-3′) [55].

### Cloning and sequencing of mutant bands

A total of 7 mutated bands amplified using genotyping assays based on TE and TE/SSR were selected for further analysis. These PCR fragments were gel purified using gel extraction kit (Qiagen). To confirm the size and purity, eluted DNA was reamplified with the specific primer and the amplified products were analyzed on agarose gels. Bands for cloning were selected based on clear pattern observed on agarose gel, reproducibility and those resulting in single amplicon upon reamplification. Purified PCR products were cloned in the plasmid pATZ57R/T using T/A cloning kit (Fermentas) and sequenced using Big Dyes terminator cycle sequencing kits (Applied Biosystems).

### Bioinformatics Analysis

Nucleotide sequences of cloned mutant fragments were used for homology searches and identification of genomic location against *Magnaporthe* genome database using BLAST analysis. Analysis of the protein sequences of genes associated with altered bands was performed. Sequence alignments were performed using ClustalW. Domain predictions and analysis were performed using Pfam, SMART, and PROSITE databases [56]–[58].

## Supporting Information

Figure S1 **Genotyping profiles obtained for** ***M. oryzae*** **control and stress treated samples using Pyret (top) and Pot2 (bottom) derived primers.** Lane 1 represent the control (untreated) sample; Lanes 2–7 represent the *M. oryzae* samples exposed to copper (0.1, 1.0 and 2.5 mM) and heat shock (1, 2 and 3 h) respectively. Lane M represents Fermentas GeneRuler 100 bp Plus DNA Ladder. Pyret based primer PyR1 (Table 1) generated variable and distinct patterns in stress exposed samples as compared to control (top). In Pot2, no altered band was observed upon stress exposure (bottom). Arrow in top gel shows copper specific band obtained using PyR1 primer.
(TIF)

Figure S2 **Dendrogram showing the clustering of control and stress exposed samples.** *M. oryzae* cultures were exposed to copper stress (Cu) and heat shock (HS). Genotyping data sets obtained using TE markers were used to generate dendrogram. *M. oryzae* exposure to copper stress (0.1, 1.0 and 2.5 mM) and heat shock (1, 2 and 3 h) resulted in induced genetic variability.
(TIF)

Figure S3 **Reverse transcriptase PCR to assess transcript levels of MINE and Pyret in control and stress treated copper (0.1, 1.0 and 2.5 mM) and heat shock (1, 2 and 3 h) samples.** Primer pairs WEIRD-125 and WEIRD-967 amplified multiple MINE transcript bands (800–1100 bp) from cDNA of stress exposed samples. The 638 bp amplicon is a fragment of the LTR-retrotransposon Pyret amplified using primers Py-FW1 and Py-RV1 [54] upon stress exposure. The 200 bp amplicon is a fragment of the *Magnaporthe* β-actin gene that was amplified as a positive control from cDNA of control and stress exposed samples.
(TIF)

Table S1 **One-way analysis of variance and Dunnett's multiple comparison test for mutant bands generated in stress exposed samples.**
(DOCX)

Table S2 **Genotyping data obtained for control and stress exposed samples using outward primers based on transposable elements.**
(DOCX)

Table S3 **Two-way analysis of variance for mutation rate of repetitive DNA obtained for** ***M. oryzae*** **stress exposed samples.**
(DOCX)

Table S4 **Two-way analysis of variance for genomic template stability indices obtained for stress exposed** ***M. oryzae*** **samples.**
(DOCX)
