# Supplementary figures and images for: Transposable Elements as Stress Adaptive Capacitors Induce Genomic Instability in Fungal Pathogen Magnaporthe oryzae
Source: PLoS One. 2014 Apr 7;9(4):e94415. doi: 10.1371/journal.pone.0094415 (PMC3978060; doi:10.1371/journal.pone.0094415)

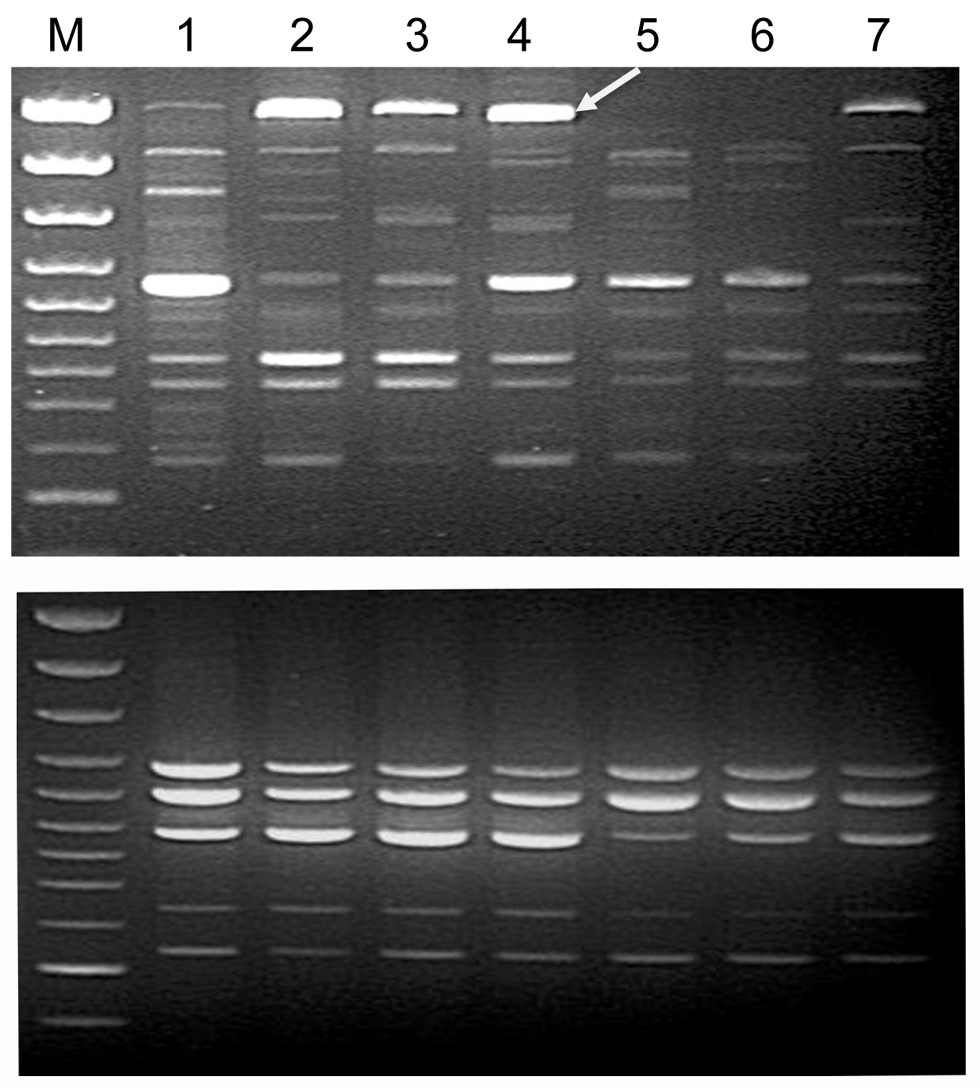

Supplement: Figure S1 — Genotyping profiles obtained for M. oryzae control and stress treated samples using Pyret (top) and Pot2 (bottom) derived primers. Lane 1 represent the control (untreated) sample; Lanes 2–7 represent the M. oryzae samples exposed to copper (0.1, 1.0 and 2.5 mM) and heat shock (1, 2 and 3 h) respectively. Lane M represents Fermentas GeneRuler 100 bp Plus DNA Ladder. Pyret based primer PyR1 (Table 1) generated variable and distinct patterns in stress exposed samples as compared to control (top). In Pot2, no altered band was observed upon stress exposure (bottom). Arrow in top gel shows copper specific band obtained using PyR1 primer. (TIF) [file pone.0094415.s001.tif]

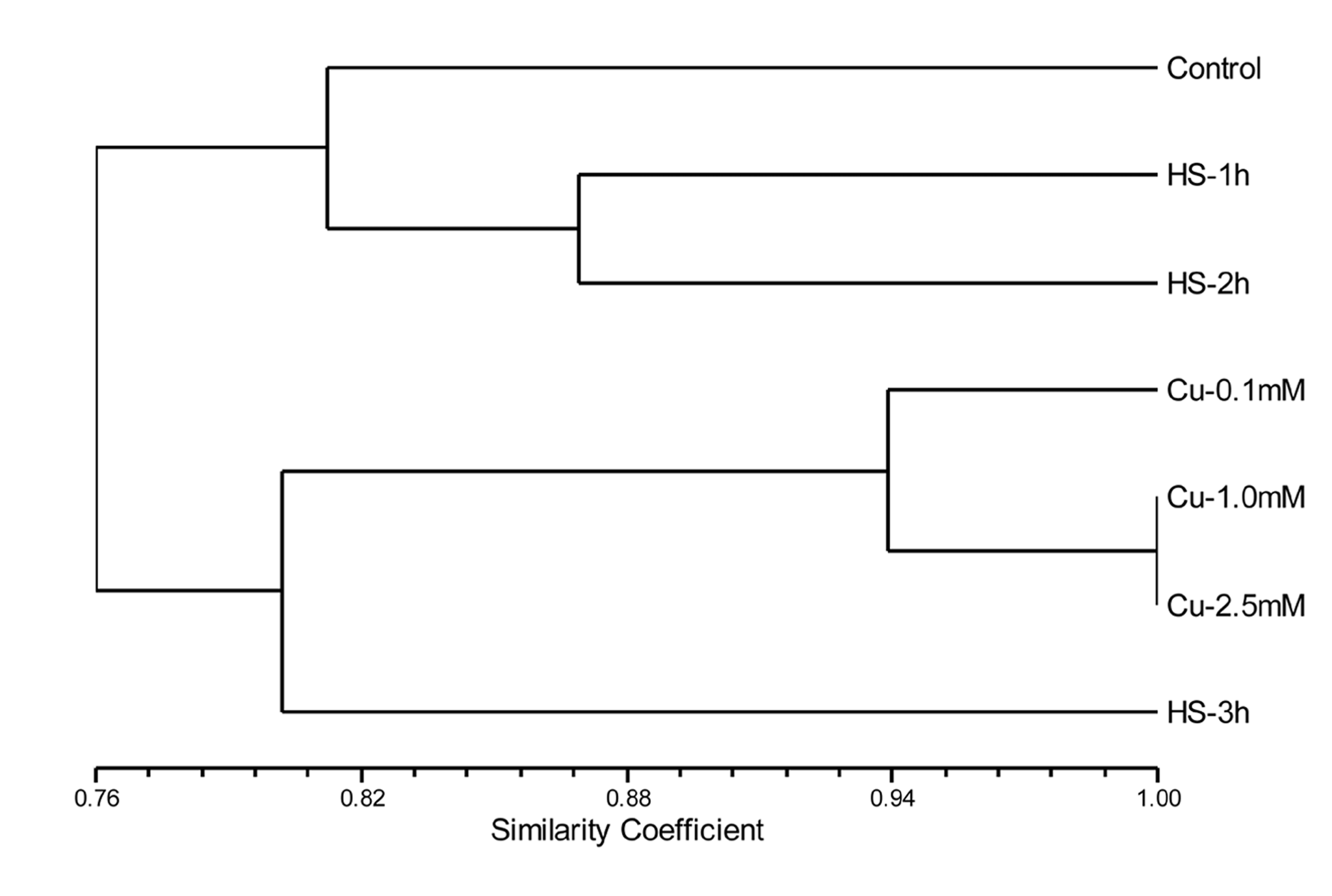

Supplement: Figure S2 — Dendrogram showing the clustering of control and stress exposed samples. M. oryzae cultures were exposed to copper stress (Cu) and heat shock (HS). Genotyping data sets obtained using TE markers were used to generate dendrogram. M. oryzae exposure to copper stress (0.1, 1.0 and 2.5 mM) and heat shock (1, 2 and 3 h) resulted in induced genetic variability. (TIF) [file pone.0094415.s002.tif]

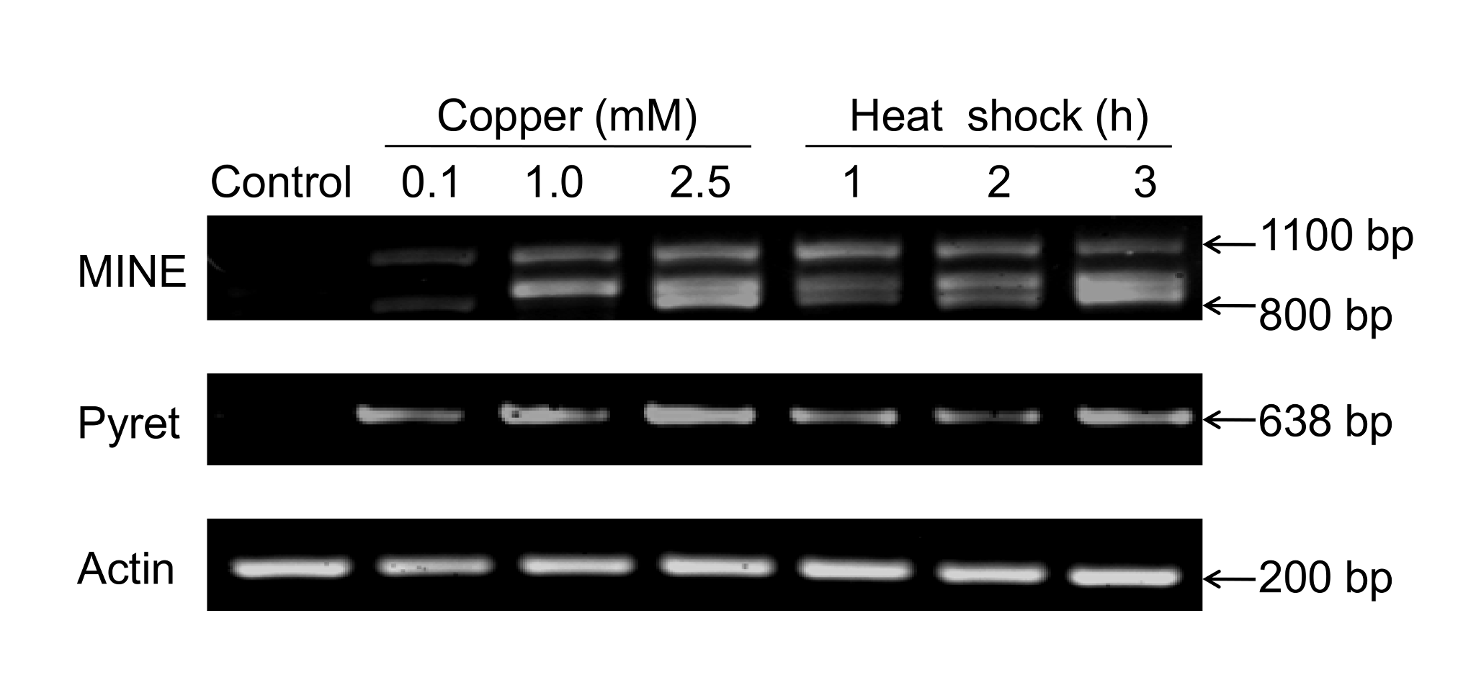

Supplement: Figure S3 — Reverse transcriptase PCR to assess transcript levels of MINE and Pyret in control and stress treated copper (0.1, 1.0 and 2.5 mM) and heat shock (1, 2 and 3 h) samples. Primer pairs WEIRD-125 and WEIRD-967 amplified multiple MINE transcript bands (800–1100 bp) from cDNA of stress exposed samples. The 638 bp amplicon is a fragment of the LTR-retrotransposon Pyret amplified using primers Py-FW1 and Py-RV1 [54] upon stress exposure. The 200 bp amplicon is a fragment of the Magnaporthe β-actin gene that was amplified as a positive control from cDNA of control and stress exposed samples. (TIF) [file pone.0094415.s003.tif]
