# Supplementary material for: Transposable Elements as Stress Adaptive Capacitors Induce Genomic Instability in Fungal Pathogen Magnaporthe oryzae
Source: PLoS One. 2014 Apr 7;9(4):e94415. doi: 10.1371/journal.pone.0094415 (PMC3978060; doi:10.1371/journal.pone.0094415)
Supplement: Table S1 — One-way analysis of variance and Dunnett's multiple comparison test for mutant bands generated in stress exposed samples. (DOCX) [file pone.0094415.s004.docx]

**Table S1.** One-way analysis of variance and Dunnett’s multiple comparison tests for mutant bands generated in stress exposed samples.

| **One-way analysis of variance** |  |  |  |  |  |
| --- | --- | --- | --- | --- | --- |
| P value | 0.0179 |  |  |  |  |
| P value summary | * |  |  |  |  |
| Are means significantly different? (P < 0.05) | Yes |  |  |  |  |
| Number of groups | 7 |  |  |  |  |
| F | 2.8653 |  |  |  |  |
| R square | 0.25973 |  |  |  |  |
|  |  |  |  |  |  |
| **ANOVA Table** | **SS** | **df** | **MS** |  |  |
| Treatment (between columns) | 103.11 | 6 | 17.185 |  |  |
| Residual (within columns) | 293.88 | 49 | 5.9974 |  |  |
| Total | 396.98 | 55 |  |  |  |
|  |  |  |  |  |  |
| **Dunnett's Multiple Comparison Test** | **Mean Diff.** | **q** | **Significant? P < 0.05?** | **Summary** | **95% CI of diff** |
| Control vs Cu-0.1mM | -2.8750 | 2.3479 | No | ns | -6.1313 to 0.38131 |
| Control vs Cu-1.0mM | -3.5000 | 2.8583 | Yes | * | -6.7563 to -0.24369 |
| Control vs Cu-2.5mM | -3.5000 | 2.8583 | Yes | * | -6.7563 to -0.24369 |
| Control vs HS-1h | -2.7500 | 2.2458 | No | ns | -6.0063 to 0.50631 |
| Control vs HS-2h | -3.5000 | 2.8583 | Yes | * | -6.7563 to -0.24369 |
| Control vs HS-3h | -4.7500 | 3.8792 | Yes | ** | -8.0063 to -1.4937 |
