# Supplementary material for: Transposable Elements as Stress Adaptive Capacitors Induce Genomic Instability in Fungal Pathogen Magnaporthe oryzae
Source: PLoS One. 2014 Apr 7;9(4):e94415. doi: 10.1371/journal.pone.0094415 (PMC3978060; doi:10.1371/journal.pone.0094415)
Supplement: Table S2 — Genotyping data obtained for control and stress exposed samples using outward primers based on transposable elements. (DOCX) [file pone.0094415.s005.docx]

**Table S2.** Genotyping data obtained for control and stress exposed samples using outward primers based on transposable elements.

| **Transposable elements** | **TNB*** | **Mutant Bands** | | | | | |
| --- | --- | --- | --- | --- | --- | --- | --- |
|  | **Control** | **Cu-0.1mM** | **Cu-1.0mM** | **Cu-2.5mM** | **HS-1h** | **HS-2h** | **HS-3h** |
| MAGGY | 7.0 | 3.0 | 3.0 | 3.0 | 3.0 | 3.0 | 4.0 |
| MGLR3 | 14.0 | 1.0 | 2.0 | 2.0 | 2.0 | 3.0 | 3.0 |
| Mg-SINE | 7.0 | 2.0 | 2.0 | 2.0 | 3.0 | 4.0 | 2.0 |
| MINE | 12.0 | 7.0 | 7.0 | 7.0 | 0.0 | 0.0 | 6.0 |
| Pot2 | 6.0 | 0.0 | 0.0 | 0.0 | 0.0 | 0.0 | 0.0 |
| Pot3 | 13.0 | 1.0 | 4.0 | 4.0 | 7.0 | 8.0 | 6.0 |
| Pyret | 12.0 | 7.0 | 7.0 | 7.0 | 6.0 | 6.0 | 5.0 |
| Grasshopper | 17.0 | 1.0 | 3.0 | 3.0 | 4.0 | 4.0 | 8.0 |

*TNB = Total number of bands
