# Supplementary material for: Transposable Elements as Stress Adaptive Capacitors Induce Genomic Instability in Fungal Pathogen Magnaporthe oryzae
Source: PLoS One. 2014 Apr 7;9(4):e94415. doi: 10.1371/journal.pone.0094415 (PMC3978060; doi:10.1371/journal.pone.0094415)
Supplement: Table S3 — Two-way analysis of variance for mutation rate of repetitive DNA obtained for M. oryzae stress exposed samples. (DOCX) [file pone.0094415.s006.docx]

**Table S3.** Two-way analysis of variance for mutation rate of repetitive DNA obtained for *M. oryzae* stress exposed samples.

| **Source of Variation** | **% of Total variation** | **P value** | **P value summary** | **Significant?** |
| --- | --- | --- | --- | --- |
| Stress * | 22.34 | 0.0001 | *** | Yes |
| Repetitive DNA ^$^ | 64.63 | < 0.0001 | *** | Yes |
|  |  |  |  |  |
| **Source of Variation** | **Df** | **Sum-of-squares** | **Mean square** | **F** |
| Stress | 1 | 257.6 | 257.6 | 31.99 |
| Repetitive DNA | 2 | 745.2 | 372.6 | 46.27 |
| Residual | 12 | 96.64 | 8.053 |  |

*Stress data: Pooled data of heat shock (1, 2 and 3 h) and copper (0.1, 1.0 and 2.5 mM).

^$^ Repetitive DNA: Pooled data for TE, SSR and TE/SSR regions
