# Supplementary material for: Transposable Elements as Stress Adaptive Capacitors Induce Genomic Instability in Fungal Pathogen Magnaporthe oryzae
Source: PLoS One. 2014 Apr 7;9(4):e94415. doi: 10.1371/journal.pone.0094415 (PMC3978060; doi:10.1371/journal.pone.0094415)
Supplement: Table S4 — Two-way analysis of variance for genomic template stability indices obtained for stress exposed M. oryzae samples. (DOCX) [file pone.0094415.s007.docx]

**Table S4.** Two-way analysis of variance for genomic template stability indices obtained for stress exposed *M. oryzae* samples

| Source of Variation | % of Total variation | P value | P value summary | Significant? |
| --- | --- | --- | --- | --- |
| Interaction | 25.30 | < 0.0001 | *** | Yes |
| Stress* | 42.73 | < 0.0001 | *** | Yes |
| Transposable elements | 22.95 | < 0.0001 | *** | Yes |
|  |  |  |  |  |
| Source of Variation | Df | Sum-of-squares | Mean square | F |
| Interaction | 14 | 9137 | 652.7 | 9.620 |
| Stress* | 2 | 15430 | 7715 | 113.7 |
| Transposable elements | 7 | 8286 | 1184 | 17.45 |
| Residual | 48 | 3257 | 67.85 |  |

*Stress data: Pooled data of heat shock (1, 2 and 3 h) and copper (0.1, 1.0 and 2.5 mM).
